# Targeting the Interplay between HDACs and DNA Damage Repair for Myeloma Therapy

**DOI:** 10.3390/ijms221910406

**Published:** 2021-09-27

**Authors:** Maria Gkotzamanidou, Elisavet Terpou, Nikolaos Kentepozidis, Evangelos Terpos

**Affiliations:** 1 Department of Oncology, 251 General Airforce Hospital, 11525 Athens, Greece; mgkotzamanidou@yahoo.com (M.G.); kentenik@hotmail.com (N.K.); 2 Department of Pharmacy, National and Kapodistrian University of Athens, 15771 Athens, Greece; elsaterpou@hotmail.com; 3 Department of Clinical Therapeutics, School of Medicine, National and Kapodistrian University of Athens, Alexandra General Hospital, 11528 Athens, Greece

**Keywords:** DNA-damage, multiple myeloma, histone deacetylases, HDAC inhibitors

## Abstract

Multiple myeloma (MM) is a malignancy of terminally differentiated plasma cells, and accounts for 10% of all hematologic malignancies and 1% of all cancers. MM is characterized by genomic instability which results from DNA damage with certain genomic rearrangements being prognostic factors for the disease and patients’ clinical response. Following genotoxic stress, the evolutionary conserved DNA damage response (DDR) is activated and, in turn, coordinates DNA repair with cell-cycle events. However, the process of carcinogenesis cannot be attributed only to the genetic alterations, but also involves epigenetic processes. Regulation of expression and activity of key players in DNA repair and checkpoint proteins are essential and mediated partly by posttranslational modifications (PTM), such as acetylation. Crosstalk between different PTMs is important for regulation of DNA repair pathways. Acetylation, which is mediated by acetyltransferases (HAT) and histone deacetylases (HDAC), not only affects gene expression through its modulation of histone tails but also has recently been implicated in regulating non-histone proteins. Currently, several HDAC inhibitors (HDACi) have been developed both in pre-clinical and clinical studies, with some of them exhibiting significant anti-MM activities. Due to reversibility of epigenetic changes during the evolutionary process of myeloma genesis, the potency of epigenetic therapies seems to be of great importance. The aim of the present paper is the summary of all data on the role of HDACi in DDR, the interference with each DNA repair mechanism and the therapeutic implications of HDACi in MM.

## 1. Introduction

Multiple myeloma (MM), an incurable hematological malignancy of plasma cells that produce a monoclonal immunoglobulin protein, is a very heterogeneous disease [1]. Despite recent advances in diagnostics and therapeutics, a subset of patients still exhibits abbreviated responses to treatment, recurrent relapses, and short survival [2,3]. Many efforts have been achieved to unravel the complex and heterogenous pathogenesis of MM and disease progression and have led to development of novel therapeutics including immunotherapies and epigenetic agents such as histone deacetylase (HDAC) inhibitors [4,5].

Histones is a group of proteins-components of chromatin and found in the eukaryotic nuclei. These proteins are grouped into five discrete classes H2A- two copies, H2B-one copy, H3 and H4. Double-stranded DNA is wound around them to form each nucleosome “core” which is the basic chromatin fibre. Between nucleosomes, a stretch of DNA—linker DNA—binds histone H1 [6,7,8]. The different lysine residues that have each histone consist one of the characteristics that differentiate these low protein molecules. Their acetylation status on lysine residues in ε amino groups is regulated by two enzymes; the histone acetylotransferases (HATs) and histone deacetylases, which catalyze the addition or the removal of the acetyl modification on lysine residues, respectively [7] and the classification of the latest is depicted in Table 1.

Changes in chromatin structure induced by HATs or HDACs lead to different access of important proteins, molecules and cellular mechanisms including transcription, protein stability and DNA damage pathways by restricting or facilitating their binding to DNA. Through these functions, the HDACs are critical regulators of gene expression with non-histone proteins and histones being targets, and the inhibition of HDACs results in various biological effects. In MM, the aberrant expression and expression of HDACs has been related to the pathogenesis of the disease and, consequently, several inhibitors are clinically tested. While these epigenetic alterations by leading to altered genetic patterns and aberrant genomic expression profiles tend to become one of the hallmarks of cancer evolution, the genomic instability remains one of the major ones. Many efforts have been undertaken to develop prognostic tools based on genomic characteristics of MM patients; it is worth mentioning that it has developed a panel of 22 DNA repair genes to assess their therapeutic role in myeloma patients included in clinical studies. Among these genes were key players of NHEJ (WHSC1, RIF1, XRCC5(KU80), PNKP, POLL), for HR (EXO1, BLM, RPA3, RAD51, MRE11A and ATM), for Fanconi Anemia mechanism (RMI1, FANCI, FANCA), for NER (PCNA, RPA3, LIG3, POLD3, ERCC4, POLD1, ERCC1 and ERCC5), and for BER (LIG3) pathways that are correlated with bad or good prognosis, independently of ISS prognostic score [9], with all the genes except ATM, POLL, ERCC5, PNKP and ERCC1, exhibiting increase expression in patients with poor prognoses.

The identification of patients that can benefit by targeted therapies or combinations of regimens with DNA-damaging agents would be of great importance. Moreover, DNA repair pathways can be deregulated in myeloma cells and modulate the activity of the already used drugs. In addition, during the evolution of myeloma from MGUS and asymptomatic smoldering myeloma, the genomic instability constitutes one of the crucial characteristics, while the underlying cause of the emergence of multiple subclones remains unclear. Malignant cells exhibit high levels of genomic instability, stalled replication forks and DSBs and, overall, impaired DNA damage response (DDR). Thus, targeting DNA repair pathways could constitute a very promising strategy to potentiate the efficacy of current regimens and reverse drug resistance, and consequently improving myeloma patients’ outcomes [10]. The maintenance of genomic stability, which is of major importance for cellular integrity, is controlled by DDR mechanisms [11,12]. Under the collective term of DDR, a sum of different inter- and intra-cellular signaling events and enzymes catalytic activities that lead to induction, detection, and repair of DNA damage in cells, is found. In case of inadequate DNA repair or suboptimal repair, the cell accumulates DNA damage and exhibits genomic instability, in that way the DDR determines the cell fate involving survival, senescence or apoptosis.

Next-generation sequencing (NGS) studies have revealed the heterogenous genomic landscape of MM with chromosomal translocations, gains or losses, hypermutation signatures and structural variations [13,14]. This genomic instability contributes to many aggressive features of myeloma disease including resistance to treatment and shorter time to relapse [15]. This genomic instability is of unknown molecular basis. The myeloma tumor microenvironment may provide an explanation for the presence of mutations and, consequently, resistance in treatment. The NGS techniques have allowed for a cost-affordable and time-effective approach of estimation or “quantification” of genomic instability in cancers [16]. Recent studies based on NGS have revealed the diversity of mutational processes underlying the carcinogenesis and novel mutational signatures in different cancer types, among them in MM [17]. HDAC inhibitors contribute to the cellular response to DNA double-strand break (DSB) formation by affecting checkpoint activation, homologous recombination–mediated repair of DNA lesions and stability of key-players enzymes [18,19,20]. Moreover, data from several studies demonstrate the therapeutic potency of combinations of HDAC inhibitors and DNA damaging agents in MM, such as SNDX-275 and melphalan or cladribine and entinostat, leading in the induction of apoptosis and the anti-proliferative effect on myeloma cells via cell cycle arrest and DNA damage response defects. In combination with the observations, the Bakkenist et al. found that chromatin changes induced by HDACi directly activate DDR mechanisms [21]. Moreover, HDACi may induce actual DNA damage directly or indirectly via increase of oxidative stress. Altogether, this leads to the conclusion of an inevitable link between the DDR and chromatin remodeling procedures via HDACs.

In this review, we aim to describe the interplay that supports the regulation of DDR in malignant plasma cells by HDACs. We also provide insight into the multiple levels of impact that HDACs exert on the induction of DNA damage and on each DNA repair mechanism focused on the identification of potent new targets and development of more effective therapeutic strategies. Finally, we enlighten the potency of combination of HDACs inhibitors that have been developed and validated in MM with DNA damaging agents, some of which are currently under study inclinical trials with promising data.

## 2. HDACs in Different DDR Mechanisms

### 2.1. Histone Deacetylases and Base Excision Repair

Base excision repair (BER) is a major repair pathway for eukaryotic cells that removes small non-bulky adducts—base lesions resulting from base oxidation induced by endogenous ROS, spontaneous deamination and alkylation induced by therapy with alkylating agents [22,23].The BER pathway seems to be crucial in the repair of melphalan-induced monoadducts [24], taking into account that melpalan retains an important role in the treatment of transplant-eligible myeloma patients.

**Figure 1:**
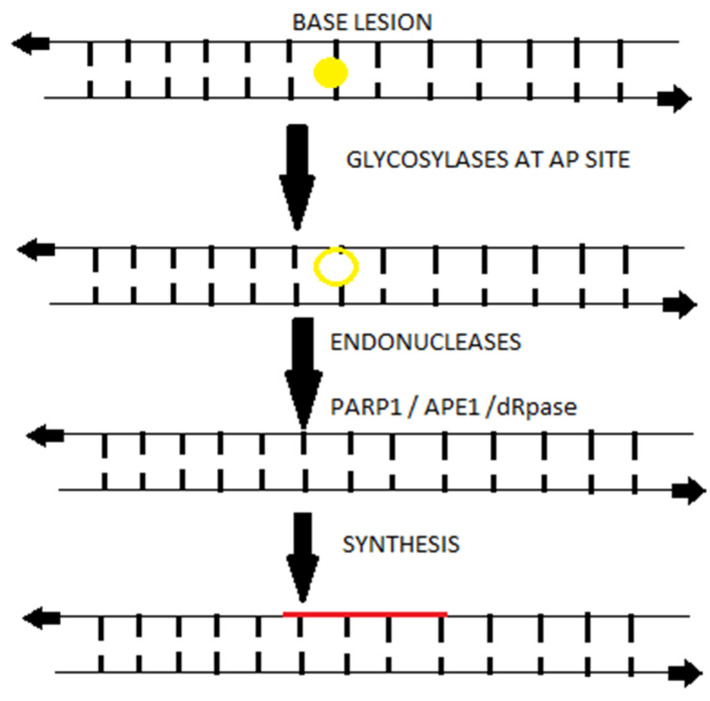
Upon a base damage occurs (yellow cycle), most often from oxidation, whereby the base excision repair is triggered and results in a short repaired a patch, single-strand segment (red line). The activity of endonucleases (yellow ring) and remove of the base are depicted at the schematic presentation of BER mechanism in steps (each long arrow shows the steps of BER mechanism).

Although the base lesions do not significantly distort the DNA helix, these can be cytotoxic by preventing the replicating polymerases during the DNA synthesis at the S phase of cell cycle; therefore it remains one of the most important repair mechanisms involving key-players molecules of other DDR mechanisms into its cataract since the DDR pathways are entwined i.e., when bulky adducts generated as repair intermediate of PARP1 can be removed by BER machinery as shown at Figure 1 [25]. Contraindicatory data from different studies have shown that genetic variants have been associated with the response of MM patients to high dose melphalan- based chemotherapy via single nucleotide polymorphisms (SNPs) in genes of the BER pathway [26,27]. Among these genes, XRCC1, a protein involved in BER and single-strand break repair, significantly resulted in the increase of accumulation of melphalan-induced DNA damage lesions in XRCC-1 –deficient myeloma cells and, in turn, sensitized them to melphalan treatment [28].

Many studies have revealed the interplay between the epigenetic modifications and the BER mechanism, with excision of 5-formylcytosine and 5-carboxylcytosine by BER the products of the epigenetic mark 5-methylcytosine [15,23].

The BER mechanism is activated by excision of the modified base via hydrolysis of the *N*-glycosylic bond which is catalyzed by DNA glycosylases that creates an apurinic/apyrimidinic site (AP site) [19]. In MM, the BER-associated AP nucleases (APEX1 and APEX2), which are involved in the recognition and repair of AP sites, contribute to the regulation of HR [29], while in experiments in myeloma cell lines it has been shown that APE1 is overexpressed in MM melphalan-resistant cells and under APE1 knock-down conditions, the melphalan resistant MM cells turned to melphalan sensitive ones [30].

On the other hand, the glycosylases that form the AP sites require various substrates and exhibit different enzymatic activity. The deacetylase SIRT1 regulates the substrate specificity of one of the DNA glycosylases—the monofunctional thymine DNA glycosylase (TDG). The deacetylation of TDG stimulates active DNA demethylation as un-acetylated TDG excises 5-formylcytosine and 5-carboxylcytosine, while acetylated TDG has a higher substrate specificity toward [31,32]. In MM the bortezomib-based treatment leads to a decrease in the SIRT1 expression; furthermore the treatment with SIRT1 resveratrol ameliorated the NALP1 upregulation and resulted in improvement of neurotoxicity-induced by bortezomib [33,34,35].

Moreover, the NAD(+)-dependent deacetylase SIRT6 was found to be highly expressed in MM cells as an adaptive response to genomic stability, and the high levels of SIRT6 were associated with adverse prognosis of myeloma patients. SIRT6 was found to interact with the transcription factor ELK1, and with the ERK signaling-related gene and the binding to their promoters and deacetylation of H3K9 at these sites, resulted to downregulation of the expression of mitogen-activated protein kinase (MAPK) pathway genes, MAPK signaling, and proliferation [36].Several studies have shown the important role of the MAPK pathway in myeloma disease with the activating MAPK pathway mutations enhancing resistance to proteasome inhibitors via increase of proteasome capacity [37,38]. SIRT6 levels increase DNA repair via Chk1 and also contribute to resistance to DNA-damaging agents such as bendamustine and melphalan [36,39,40].

The role of SIRTs in the BER mechanism, however, is not limited to the previous mentioned contribution. The excision of the false bases by glycosylases is followed by the binding of AP sites and single-strand breaks (SSBs) by poly ADP ribose polymerase 1 (PARP1), which changes its structure by negatively charging its PAR chains and allowing the recruitment of key proteins of BER. Moreover, SIRT6 activates PARP1 and contributes to cell fate since it protects against base lesions that can tolerate the cells.

In addition, SIRT1 associates with the apurinic/apyrimidinic endonuclease 1 (APE1) by targeting lysines 6 and 7 of, stimulating its endonuclease activity and finally protecting cells against cytotoxic base lesions methyl methanesulfonate (MMS) –induced or H2O2. SIRT1 also deacetylases the RecQ protein Werner (WRN) with helicase and exonuclease activity [40,41]. The deacetylation of WRN leads to stimulation of POL-β to insert new nucleotides, while in case of modifications i.e., oxidations or reduction on 5′end other polymerases (δ or ε), the POL-β inserts up to 10 nucleotides. The deacetylation of WRN by SIRT1 stimulates its exonuclease activity as well, regulating in different levels the efficacy of the BER mechanism [41,42].

### 2.2. Histone Deacetylases in Nucleotide Excision Repair Mechanism

**Figure 2:**
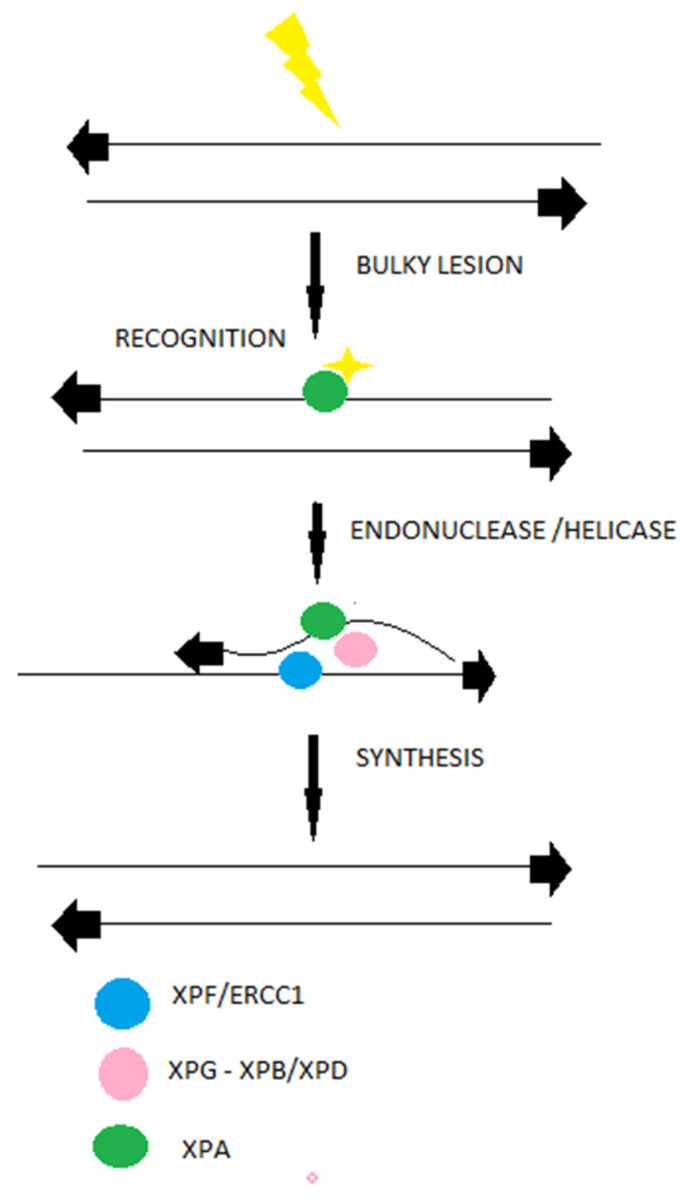
Bulky nucleotide lesions (most often from ultraviolet light–depicted as yellow symbols) are repaired by NER. This machinery involves a long-repaired single-strand patch as shown in the different steps of NER machinery (long arrows).

The nucleotide excision repair mechanism (NER) is one of the important DDR repair mechanisms that removes around 30 nucleotides surrounding a DNA helix-distorting lesion [43]. NER recognizes bulky lesions induced by ultraviolet light, adducts formed by epoxide intermediates during detoxification of polycyclic aromatic hydrocarbons from tobacco or food or alkylating agents such as melphalan. following the recognition, with hRAD23B and centrin 2 (CETN2) constitute a complex [43,44]. There are types of lesions that do not destabilize the DNA dublexes and are first recognized by DDB2 (XPE) in complex with DDB1, and consequently are recognized by XPC. Altogether, the XPC-hRAD23b-CETN2 complex melts the DNA around the lesion and recruits the transcription factor IIH (TFIIH) and its subunits XPB and XPD, as shown in Figure 2 [45]. The last two proteins participate in DNA strand opening and unwinding, stabilization, and assembly of the XPG endonuclease responsible for the 3′ incision and ERCC1-XPF (excision repair cross-complementation group 1), which is responsible for the 5′ incision. A recent study revealed that cell lines with high NER activity tend to show resistance to melphalan, and that NER activity varies in patients with MM [46]. By utilizing gene-expression profiling (GEP), Cuce and colleagues evaluated the expression of NER genes and, consequently, their impact on apoptosis, cell cycle and other cellular functions in MM cell lines and patient-derived primary MM cells exposed to increasing nanomolar concentrations of trabectedin [47]. In normal compared to MM plasma cells (PCs), an enrichment of DNA NER genes in poor prognosis for the disease of MM was observed. Among these genes, only four (XPA, RAD23B, XAB2, and POLD3) showed independent predictive power. A significantly high expression of RAD23B, XAB2, and POLD3 was associated with poor prognosis, whereas XPA was associated with more favorable survival rates [47]. Moreover, overexpression of excision repair cross-complementation group 3 (ERCC3) leads to increase of resistance to melphalan, while the ERCC3 knockdown cells exhibit decreased NER efficacy and high sensitivity to melphalan, underlining the important role of NER mechanism in resistance of myeloma cells to alkylating agents [46]. Spironolactone, a small molecule, has been found to inhibit the NER efficiency and, in turn, to revert the resistance of myeloma cells to melphalan [48].

The NER efficacy is modified by epigenetic modifiers in cancer cells. The deacetylase activity of SIRT1 stimulates the expression of XPC. In this way, the SIRT1 contributes to initiation of the GG-NER pathway by XPC and confers resistance of cancer cells to platinum drugs, UV light or cross-linking agents [49,50].

Moreover, the nuclear SIRT1 stimulates the recognition of the lesion by promoting the overexpression of XPC. It stimulates the lesion excision via enhancing the assembly of endonucleases’ complex at the lesion by deacetylating the XPA [49]. In addition, the overexpression of HDACs in different cancer cells also contributes to activation of the NER mechanism in many different types of cancer, MM amont them, as HDAC inhibition (especially of classes I and II) leads to inhibition of the removal of bulky lesions by key-players of the NER pathway [51,52,53].

### 2.3. Histone Deacetylases in Non-Homologous End Joining Repair Mechanism

**Figure 3:**
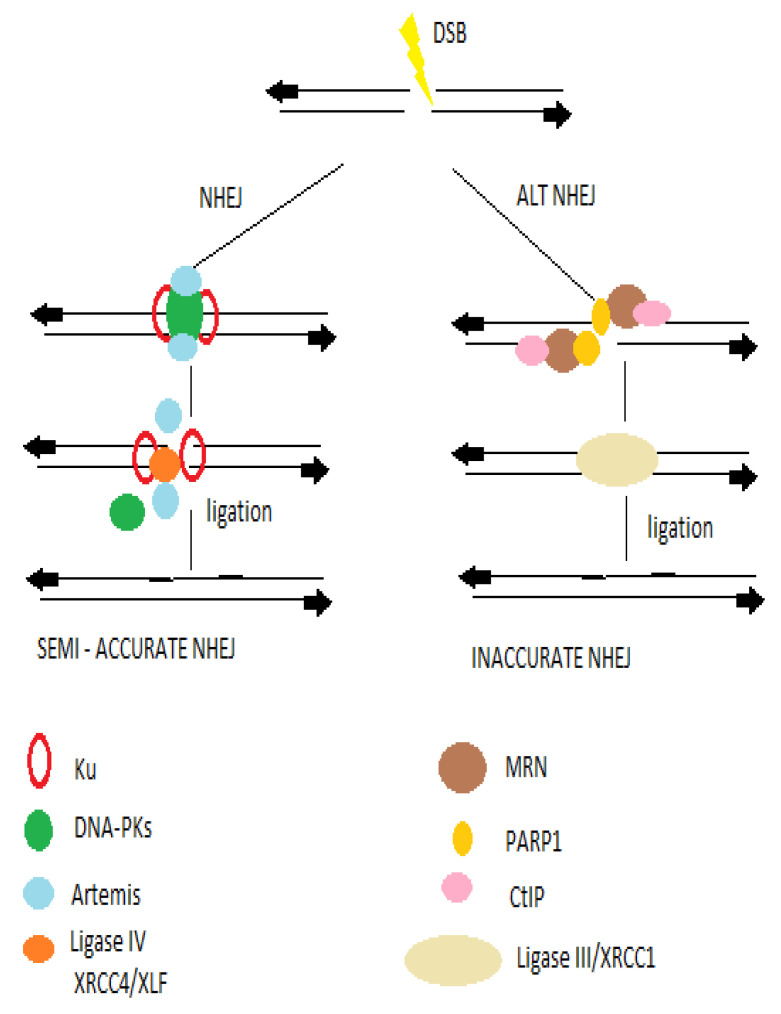
Upon a DSB occurs, the NHEJ and HR sub-pathways are involved, depending on the homology of the ends of the DSB. The NHEJ includes the classic and the error- prone alternative NHEJ pathways. The classic NHEJ pathway initiates with broken ends bound by Ku proteins, which protects ends. Following the Ku binding a cataract of endonucleases and ligases, key-players of NHEJ is taking place.

The non-homologous end joining repair pathway could be mainly divided into three distinct steps: the recognition of DSBs, the processing, and the ligation, while the ends of DSBs define the participation of more factors and further procedures into the ligation [54,55] as shown in Figure 3. Among the key-players of NEHJ are the Ku70/80heterodimer, XRCC4, the DNA-dependent protein kinase catalytic subunit (DNA-PKcs), XLF (XRCC4-like factor), and DNA ligase IV and Artemis [56].

The repair of ionizing radiation- or RAG-induced DSBs during the V(D)J recombination, which occurs in chromatin centrally involves the chromatin-modifying enzymes.

In MM the activity of both NHEJ and HR was elevated in MM cells in comparison to healthy controls, while key-molecules such as DNA ligase IIIa were involved in alternative NHEJ pathways that promote to more mutagenic-prone cells. [57]. Furthermore, in MM suppression of aberrant NHEJ function by using NU7026, a DNA-PK inhibitor might facilitate access of DNA ends to an intact HR pathway and lead to increase of survival of myeloma cells post irradiation, suggesting that deregulation of the NHEJ repair pathway may contribute to genomic instability and clastogenic stimuli during evolution of myeloma disease. Moreover, MM cell lines studied in the same study were variably impaired in both DNA rejoining efficacy and fidelity [58].

In addition, genetic variants of important molecules of NHEJ, such as DNA ligase IV have been identified in myeloma cells, indicating that these might modulate the predisposition to symptomatic disease. The polymorphisms LIG4 A3V CT, T9I CT and the T9I TT have been significantly associated with a two-fold reduction of risk of multiple disease [59] and is accosiated with antigen receptor rearrangement and maintenance of genomic stability efficiency [60], while other key-players i.e., XRCC4 and RAD50 were found overexpressed in MM. A small-scale study revealed that in 15 out of 16 MM cell lines, lack of Ku80 alterations at the protein, mRNA and gene level was observed, while no aberrant Ku80 was observed in only six patient samples [61].

The efficiency of NHEJ is under epigenetic regulation, as well as other repair pathways. Hdac1 and hdac2 by deacetylating the H3K56Ac and H4K16Ac leads to stimulation of NHEJ repair pathway [56]. Moreover, in absence of Sirt7 in vivo experiments with xenograft models showed that the consequent increase of H3K18 acetylation led to an inefficient NHEJ mechanism [62,63].

As mentioned above, the Ku70/80 heterodimer plays an important role in the NHEJ by initiating the cataract after its binding to DSB ends, and in that way protects them from degradation by exonucleases. The heterodimer also stabilizes anti-apoptotic c-FLIP and proapoptotic Bax proteins, which are regulated by acetylation. Therefore, HDAC inhibition induces Ku70 acetylation with repressed c-FLIP and activated Bax in different cancer cells, making the Ku70/80 complex a possible target of HDAC inhibitors [64].

Current studies have shown that the NHEJ pathway in myeloma is aberrant, with up-regulation of gene expression of NHEJ key-players (including ku70, and ku86) to be related to poor prognosis of myeloma patients [58]. In myeloma cells, the Ku86 variant was found to be involved in decreased DNA repair efficacy and to confer increased sensitivity to radiation and to DNA damaging agents. HDAC 1, 2 and 3 are shown to play an important role in the deacetylation of ku70, with inhibition of HDAC 1, 2 and 3 resulting in increased ku70 acetylation, decreased Ku70 binding on DSB, and increased sensitivity to DNA damaging agents. In addition, the interplay between the ku70/80 heterodimer and DNA-PKcs activates the activity of kinase of the complex, which leads to autophosphorylation of DNA-PKcs and the following phosphorylation of NHEJ factors [65].

On the other hand, class III of histone deacetylases, Sirtuins and especially SIRT6 stabilizes and promotes the localization DNA-PKcs at the site of the DSB, while SIRT1 deacetylates Ku70 and TIP60, KAP1 and HDAC1 and, consequently, stimulates HDAC1 activity and NEHJ efficacy, based on studies in mammalian cells (Hela cells) [66,67]. Furthermore, in the absence of SIRT7 in mice an increase of H3K18Ac levels was observed that affected the NHEJ efficiency, as observed in different cancer cell lines (Hela & U251 cells) [68,69].

### 2.4. Histone Deacetylases in Homologous Recombination in Myeloma

The homologous recombination (HR) repair mechanism is essential to access the redundancy of genetic information as formed as sister chromatids. The main role of HR is the control of DNA replication without errors via repair of DSBs during the process of meiosis. The HR repair mechanism requires homologous sequences on sister chromatids and is active in the late S or G2 phases of the cell cycle [69].

**Figure 4:**
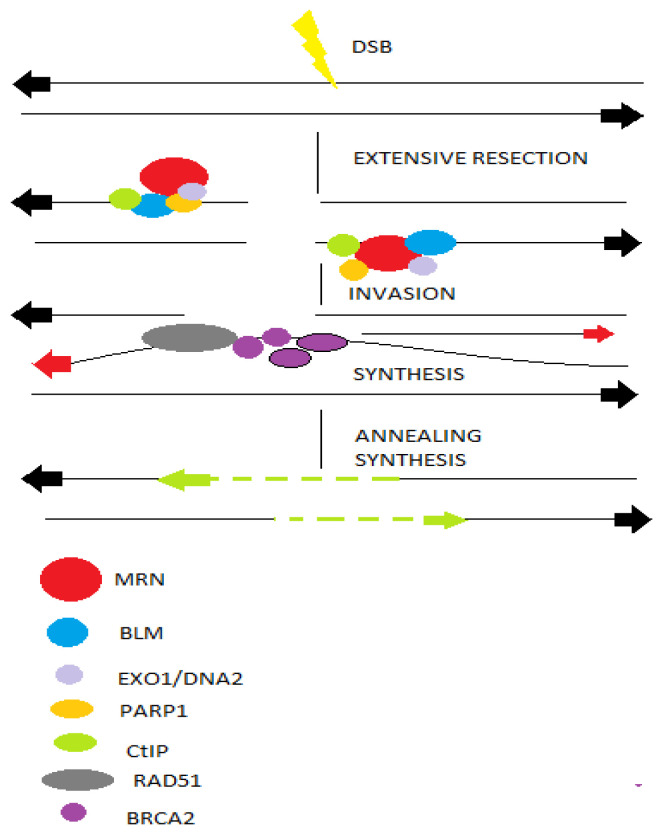
DSBs occur in transcriptionally active or replicating cells due to reactive oxygen species or ionizing radiation or during replication process. The choice between HR or NHEJ is directed by BRCA1 and 53bp1. Moreover, PARP1 promotes more extensive end-resection via exonuclease activity of EXO1 and BLM to reveal ssDNA and promote HR. RPA binds to ssDNA, while the BRCA2 mediates replacement of RPA with RAD51. Finally, the RAD51 nucleoprotein filament invades a homologous sequence, acting as donor and synthesis extends the invading 3′ end, which then anneals with the resected end to lead to accurate repair.

The HR pathway begins with nucleolytic resection of DNA ends, which is mediated by Mre11-Rad50-Nbs1 (MRN)complex, breast cancer susceptibility (BRCA1) and CtIP. The key-players of HR mechanism yield 3′ single-stranded DNA tails that are stabilized by replication protein A (RPA), while CtIP promotes HR by initiating DSB end resection and the formation of ssDNA (Figure 4). Following the formation of ssDNA, BRCA2 catalyzes the displacement of RPA and the formation of the nucleoprotein RAD51, which promotes homology detection and the switch of broken strands [70].

The strand where the lesion is located is extended based on the sister chromatid, which is used as a template.

In myeloma patients, HR efficacy is higher compared with healthy donors, as evidenced in plasmid assays indicating HR activity, while genomics studies have shown an increase of genes and proteins involved in HR repair mechanisms, among them RAD50 and RAD51. Moreover, in MM cell lines, increased HR efficacy resulted to resistant cells to dexamethasone and addition of genomic instability [29,71].

The histone deacetylases mediate HR repair mechanism with multiple aspects. The CtiP is acetylated and degraded after HDAC inhibition.

Interestingly, the SIRT6 has been implicated in acetylation of CtIP, and therefore in absence of SIRT6 cancer cells are more sensitive to DNA damaging agents and PARP inhibitors [72]. SIRT6 promotes end-resection and in conditions of depletion of SIRT6 the RPA which binds the ssDNA ends, is less efficient [73,74].

Moreover, in hematologic malignancies, it has been shown that HDAC3 is necessary for the resolution of replication-coupled errors, while inhibition of HDAC3 resulted in decrease of the efficacy of cellular repair mechanisms against replication stress.

However, the impact of deacetylation on HR is not limited only on CtIP. SIRT1 deacetylates NBS1 as well [75]. The pan-HDACi SAHA resulted to downregulation of RAD50 and MRE11, following by a decrease of HR activity in cancer cells [76].

Other key-players of HR, among them RAD51 and its paralogs RAD51B, RAD51C, RAD51D, and SIRT6 exhibit decreased expression if cells increase the replication cycles, with overexpression of SIRT6 to cancer cells, and in myeloma cells as well, to contribute to increased HR repair activity and, consequently, the addition of genomic instability to them.

Additionally, the inhibition of class I HDACs by MS-275 or in cancer cells HDAC2 knockdown, including osteosarcoma, prostate cancer- melanoma- and myeloma-cells, resulted to a decrease in RAD51 expression and dysfunctional HR repair mechanisms [77,78].

On the other hand, in HeLa cells the knockdown of HDAC9 or HDAC10 led also to decreased HR efficacy and sensitivity of cells to DNA interstrand crosslinks-inducing agents such as mitomycin, indicating that HR repair mechanism coordinates with other major repair mechanisms for the continuous cellular protection [79,80,81].

### 2.5. HDACs and Repair of Interstrand Cross-Links in MM

One of the most toxic form of DNA lesions which prevent transcription and replication via inhibition of DNA strand separation are interstrand cross-links (ICLs).

The presence of even a single ICL in the eukaryotic genome can cause severe defects in a variety of vital DNA metabolic processes, while ICLs accumulation over time contributes to genomic instability and, consequently, to cancer evolution and aging processes [82]. The ICL repair mechanism involves the Fanconi anemia proteins, the gap-filling Translesion DNA Synthesis (TLS) polymerases which replicate the past DNA lesions and the key-players factors of NER and HR repair pathways.

These lesions are formed in the presence of bifunctional alkylating agents, some of which are still most widely used in treatment of multiple myeloma such as melphalan, which is used as a preparative agent to autologous stem cell transplantation to eligible MM patients. Spanswick et al. showed that sensitivity to melphalan in plasma cells from naive and melphalan-treated patients, in myeloma cell lines as well, were correlated with ICL repair [82].

In one of our previous studies, it has been shown that newly diagnosed MM patients who were characterized by slower rates of NER and DSB repair mechanisms (resulting in higher accumulation of the highly cytotoxic ICLs and DSBs lesions), responded to first-line treatment with melphalan and exhibited improved clinical outcomes [83].

ICLs are detected during any phase of the cell cycle affecting both DNA strands, by the NER protein XPC in the crosslink that distorts the DNA or by CSB [84]. Incision of the ICL lesion could occur independently of bypass, resulting in a DSB subject to HR or NHEJ mechanisms. The mammalian repair of ICLs differs from yeast or *E. Coli* repair mechanisms. The loss of Excision repair cross complementing gene 1 (ERCC1) or xeroderma pigmentosum group F (XPF) proteins in knocked-down cells, forms a heterodimer that results to enhancement of sensitivity to ICLs of these cells However, many other proteins might be involved in this process [85,86]. The ICL repair follows the next steps of DSB formation, resection of the lesions and invasion of the DNA stands, with the final steps of end trimming and increase of activity of FA pathway.

HDACs play an important role by participating in different steps of ICL repair.

As above mentioned the NER- dependent removal of ICL lesions as sodium butyrate leads to a decrease in the repair efficacy of UVA-induced ICLs [87]. Moreover, SIRT1 facilitates the detection of ICLs by stimulating the XPC, which might lead to resistant cancer cells to ICL-inducers agents such as fotemustine [88,89]. ERCC-XPF incises close to the spot of the ICL and unhooks it, following by filling the sequence with translesion synthesis, while the SIRT1 increases the interaction of XPA with the complex of ERCC-XPF [49]. On the other hand, the usage of specific SIRT1 inhibitor EX-527 confirmed that SIRT1 deacetylases XPA in cancer cells [90], while treatment of MM cells with SRT1720, another SIRT1 inhibitor, resulted in inhibition of cellular growth and induction of apoptosis in MM cells resistant to conventional and bortezomib therapies without any significant effect on the viability of normal cells [35].

Moreover, in comparison to acetyl-XPA, the deacetylated XPA by SIRT1 exhibits stronger binding to chromatin and, consequently, triggers NER more effectively; the acetylated XPA may hence increase the persistence of ICL lesions and improve the antitumor activity of cross-linking agents [90].

These DNA cross-linking agents such as melphalan are still important in the treatment of multiple myeloma and of other cancers. Chen and his colleagues showed that the overexpression of the FA/BRCA pathway genes contributes to acquired resistance to melphalan in myeloma cell lines. Moreover, the FA/BRCA pathway contributes to drug resistance via enhanced ICL repair and release of cells from melphalan-induced growth inhibition [91]. The proteins of the FA pathway participate in the ICL repair by contributing to the initiation of replication-dependent repair. FANCM recognizes the replication fork and leads to its regression in a chicken foot-like DNA structure. The heterodimers of FANCD2 and FANC1 will be monoubiquitinated by the FNACL subunit of the FA complex [92]. The FANCD2 heterodimer recruits other nucleases, among them SLX4-SLX1, MUS81, and ERCC1-XPF to enhance the incision and the uncoupling of the ICL. In addition, the effect of HDAC inhibitor MS-275 leads to a transcription downregulation of FANCD2 and increase in the sensitivity of cancer cells to fotemustine [77].

The pan-HDACi SAHA and trichostatin A that have been clinically tested in MM, augment the histone acetylation at binding sites of the epigenetic silencing factor enhancer of zeste-2 homolog 2 (EZH2) which consequently suppresses the Xeroderma pigmentosum complementation group A (XPA)gene in multiple cancer cells [93,94]. There is a molecularly defined interplay between EZH2 and BRCA1 with reduced levels of BRCA1 leading to an altered localization of EZH2, an increase of histone H3 trimethylation at K27, and, consequently, an increase of metastatic potency in cancer cells [95].

While the unhooking of ICLs results in a one-ended DSB in the excised strand and replication fork remodeling as mentioned above, upon recognition of these DSBs by ataxia telangiectasia mutated (ATM), these are processed by MRN complex and activation of further steps of the HR repair mechanism, including the CtIP, BRCA1, and EXO1. The HDACs regulate the acetylation of EXO1, RAD50 and CtIP. The CtIP is necessary for the accumulation of key-players proteins of ICL repair mechanism such as the checkpoint kinases ATM- and RAD3-related, ATR and ATM with their targets Replication Protein A (RPA) and H2AX/ FANCD2, respectively [72,96]. These BRCA1-mediated activities of CtIP harbor the ICLs and are dispensable for the activation of ATM and H2AX upon generation of DSBs.

The deacetylation of CtIP by SIRT6 significantly controls DSB resection. Furthermore, the regulation of HR repair by SIRT1 and 6, HDAC9 and 10 crucially contributes to HR-mediated fork restart, with preclinical studies with knockdown models of HDAC9 and 10 underlying the sensitivity of these cells to mitomycin [80]. The fork restarts and the uncoupled ICL being removed by NER proteins while the ICLs block the DNA and RNA polymerases. The TC-NER mechanism contributes to the DNA replication-independent repair of ICLs induced by UV or cisplatin [97,98]. The recruitment of HDAC1 and DNMT3b has an impact on regulation of ICL repair, while the MS-275 has been found to increase the CSB levels, and all together lead to decrease of important GG-NER proteins. Therefore, the impact of HDAC on ICL repair could occur in multiple ways, one of which is the direct pathway choice between the GG-NER and TC-NER.

## 3. Conclusions

One of the hallmarks of cancer is the genomic instability that mainly results from DNA damage [99]. Therefore, eukaryotic cells have developed control mechanisms which maintain their genome integrity and repair possible DNA damage [100]. Major characteristics of MM are genomic instability, stalled replication forks, and DSBs. The MM complex genomic landscape is characterized by chromosomal gains or losses, structural variations, and cancer-driver mutations [101,102]. These lesions occur through various forms of genomic instability such as microsatellite instability and chromosomal instability, but also, during the process of myeloma genesis, from monoclonal gammopathy of undetermined significance (MGUS) and asymptomatic myeloma (SMM) to symptomatic MM [103,104]. Under natural circumstances, DNA is constantly exposed to numerous risk factors from either endogenous metabolic process, such as reactive oxygen species or exogenous sources including UV light, chemical agents, ionizing radiation, and others. MM patients exhibit higher levels of endogenous DNA damage, as previous studies have shown [105,106]. In all these lesions, the evolutionary conserved DDR is activated to detect, process, and correct most of them, in order to prevent chromosomal rearrangements. The form of chromatic structure is one of the multiple defensive tools that the cell possesses, and this and contributes to effective DDR. Epigenetics—the heritable alterations in expression of genes that are not accompanied by changes in sequence of DNA, orchestrate the changes in chromatin structure and, consequently, participate in various important cellular processes such as differentiation and cell cycle [107]. The complexity of the interplay of HDACs on the DDR-related proteins could be described with the regulation of p53—the guardian of the genome. The tumor suppressor protein p53, which plays a critical role in maintenance of genomic stability, and its mutations lead to cancer susceptibility, and this regulates upon DNA lesions the cell fate by promoting the transcription of genes that are involved in critical cellular processes such as apoptosis, cell cycle and senescence [108,109]. In the absence of stress, the levels of p53 remain low under the E3 ubiquitin ligase MDM2 regulation. Upon DNA damage, the p53 gets phosphorylated by DDR kinases ATM, ATR, Chk1 and Chk2that are also direct targets of HDAC1 and SIRT1, as mentioned above (Table 2) [110]. Consequently, the phosphorylation of p53 results to disruption of the p53-MDM2 interaction and to accumulation of p53.

Following the phosphorylation, the phosphorylated p53 is further activated by acetylation. The acetylation of p53, as with the majority of histone modifications, is a reversible process, and it has been shown that MDM2 recruits HDAC1 to form a complex that leads, in turn, to deacetylation of p53.

The most important lysine-acetyl-transferase that is involved in the p53 activation is p300/CBP. P300 acetylases the p53 in several residues, among them the lysine-382 (K382), while p300 constitutes a direct target of SIRT1as well.

Previous studies have shown that p300 can also form a complex with MDM2 that facilitates the degradation of the p53 protein [111]. Vice versa, the expression of SIRT1 and its deacetylase activity are regulated in normal cells by multiple proteins among them the p53, the cell cycle apoptosis regulator 2, the Chk2, the testis-specific protein Y-encoded-like 2 (TSPYL2) [112]. Furthermore, the p300 activity toward p53 is negatively regulated by the SIRT1 that deacetylates the p53, resulting in prevention of p53-dependent transcription and cellular apoptosis [113]. The underlying mechanisms of this ambiguous regulation are not fully understood. The SIRT1 and p53 proteins are NAD+ dependent. NAD+ as co-factor allows the binding to p53 tetramers and modifies the binding efficiency to p53 and in that way, leads to decrease of p53-mediated transcriptional activity with major effect on cell cycle arrest, apoptosis and enhancement of DDR mechanisms [114]. All the above suggest that the HDAC-DDR axis can modulate the fate of somatic and stem cells with the final phenotype of genomic instability, aging and cancer, while the SIRT1-p53 axis constitutes just one example of this interaction with multiple therapeutic and prognostic implications in oncology.

**Figure 5:**
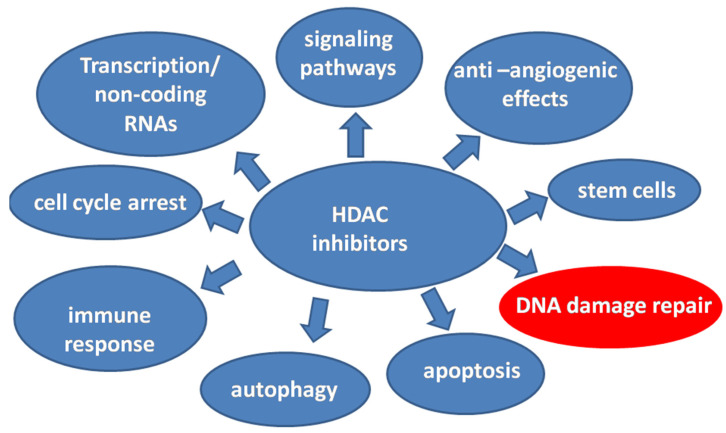
The different functions of HDAC inhibitors in cancer. The interplay of histone deacetylation inhibition and DNA damage in MM becomes a very promising therapeutic strategy.

Much recent work indicates that genetics and epigenetics cooperate at all stages of cancer evolution with one major epigenetic alteration-the histone acetylation. HATs and HDACs have a concerted action of histone acetylation by adding or removing acetyl-groups, respectively, on lysine residues and, consequently, this leads to remodeling of the chromatin structure. However, it has been shown that HDACs and SIRTs could deacetylate and other non-histone substrates modifying their action. Therefore, the targets of HDACs become important key-players in cellular biology with multiple functions and mode of action and consequently, the HDACs a very promising therapeutic strategy in multiple myeloma [33,36,115,116]. Some of the different functions of HDAC inhibitors in cancer are depicted in Figure 5. Therefore, in 2015 the FDA approved the first HDAC inhibitor, Panobinostat, in combination with bortezomib and dexamethasone for patients with relapsed or refractory myeloma [117]. Thereafter, the HDAC inhibitors are undergoing extensive preclinical and clinical evaluation as single agents or in combination with other anti-myeloma agents.

Changes in chromatin structure induced by HDACs inhibitors activate DDR and have an impact on efficacy of different repair mechanisms including HR and NHEJ, as analyzed above and depicted at Table 2. These observations lead to the conclusion that HDAC inhibitors have synergistic effect on cytotoxicity and growth inhibition of myeloma cells. Many current clinical trials (Table 3) evaluate the efficacy of ACY-1251, belinostat, AR-42 and others in combination with in IMIDs, mTOR inhibitors and DNA damaging agents in relapsed and/ or refractory MM [117,118,119,120]. The rapidly growing armamentarium of myeloma therapeutics makes the mechanism-based targeted therapies more than necessary. The understanding of underlying mechanisms of action of HDACs inhibitors and their interplay with DNA damage repair of keymolecules will contribute significantly, from a therapeutic perspective, to the development of more effective myeloma treatments.

We have sought, in the present review, to revisit, refine, and extend the concept of interplay between HDACs and DNA damage repair mechanisms, which could provide a useful conceptual framework for understanding the complex myeloma biology and therapeutic approach of disease.

In conclusion, the future of therapeutics in MM relies on combinational therapies and individualized therapy based on genetic and epigenetic profiles-two of the cancer hallmark capabilities. Further mechanistic studies are needed to elucidate the histone deacetylations multiple effects on cellular biology in order to improve the in vivo pharmacokinetics of HDACs inhibitors, to eliminate the side effects of them, and, finally, to identify the subpopulation of myeloma patients that could benefit from combinational therapies.

## Figures and Tables

**Table 1:** The different classes of HDACs, the multiple DDR key-components and the specific DDR cellular pathways as their targets.

Class of Deacetylases		Impact on Components of DDR Pathways	DNA Damage Pathway
Class I	HDAC1 HDAC2 HDAC3 HDAC8	*p*-Glycoprotein, RAD51 TIP60	NHEJ, HR
Class IIA	HDAC4 HDAC5 HDAC7		
Class IIB	HDAC6		
Class III	SIRT1 SIRT6 SIRT7	APE1, WRN, PARP1, NBS1, DNA-PKs, CtIP, Ku70, XPA Ku70/ Ku80, BRCA1, Rad50, Rad51, DNA-PKcs, mre11 FANCD2, CHK1	NER, HR, NHEJ

**Table 2:** The impact of HDACs on each DDR mechanism and their targets. The last column shows some of the selective HDACs inhibitors that are used in pre- and clinical setting in cancers.

	DDR Components	Cellular Procedures—DDR	HDAC Inhibitors
HDAC1	ATM, ATR, H3K56, H4K16, P53, Ku70, APE1/Ref1, CtIP	NHEJ HR Chromatin remodeling Apoptosis	MS-275 CBUD-1001
HDAC2	H3K56, H4K16, Ku70	apoptosis chromatin remodeling	
HDAC3	H3K9 and K14, H4K5 and K12	Chromatin remodeling	
HDAC4	53BP1		
HDAC6	Ku70 GADD153	Apoptosis	ACY-1215 ACY-241
SIRT1	H3K9, H4K16, P53, NBS1, XPA, Ku70, FOXO, WRN, XPC, RelA/P65,APE1/Ref1, TIP60, DNMT1, P300	NER BER NHEJ HR Chromatin remodeling apoptosis	
SIRT3	H4K16, Idh2	Chromatin remodeling oxidative stress	
SIRT6	CtIP, XPA DNA PKs H3K9, H3K56	HR BER NHEJ Chromatin remodeling	OSS-128167

**Table 3:** The current active and recruiting clinical trials of HDACs inhibitors in multiple myeloma. Accessed on 2 September 2021 [120].

Study	Status	Study Results	Conditions	Characteristics
A study of PVX-410, a cancer vaccine, and Citarinostat +/− Lenalidomide for Smoldering MM	Recruiting	No Results Available	Smoldering Multiple Myeloma	Phase 1
Study of ACY-1215 in combination with pomalidomide and dexamethasone in Multiple Myeloma	Active, not recruiting	No Results Available	Multiple Myeloma	Phase 1 Phase 2
Study of ACY-241 Alone and in combination with pomalidomide and dexamethasone in Multiple Myeloma	Active, not recruiting	No Results Available	Multiple Myeloma	Phase 1
A study of HG146 Capsule in Chinese Subjects with Relapsed and Refractory Multiple Myeloma	Recruiting	No Results Available	Multiple Myeloma Relapsed and Refractory Multiple Myeloma	Phase 1
Study of Tinostamustine, First-in-Class Alkylating HDACi Fusion Molecule, in Relapsed/Refractory Hematologic Malignancies	Recruiting	No Results Available	Hematological Malignancies Multiple Myeloma Hodgkin’s Lymphoma Cutaneous T Cell Lymphoma	Phase 1
Pralatrexate + Romidepsin in Relapsed/Refractory Lymphoid Malignancies	Recruiting	No Results Available	Lymphoid Malignancies Multiple Myeloma Lymphoma Hodgkin Lymphoma Non-Hodgkin Lymphoma	Phase 1 Phase 2
A disease Registry Encompassing the care of patients with Multiple Myeloma on Panobinostat	Recruiting	No Results Available	Multiple Myeloma	
Panobinostat (LBH589) Multiple Myeloma-Autologous Hematopoietic Cell Transplantation (HCT)	Active, not recruiting	Results available	Multiple Myeloma	Phase 2

## Data Availability

Not applicable.
